# Bromoderma caused by over-the-counter analgesic mimicking pyoderma gangrenosum

**DOI:** 10.1186/2045-7022-4-S3-P73

**Published:** 2014-07-18

**Authors:** Fumiko Oda, Mikiko Tohyama, Koji Sayama

**Affiliations:** 1 Ehime University Graduate Scool of Medicine, Dermatology, Japan

## 

A 41-year-old Japanese woman was admitted to the emergency department of our hospital due to a seizure. She had been diagnosed with pyoderma gangrenosum 11 years ago without bone marrow disease, rheumatoid arthritis, or inflammatory bowel disease. Pyoderma with painful ulcers had repeatedly appeared on the arms, legs, face, and pubic arch despite treatment with systemic prednisolone and cyclosporine. She also had frequently complained about syncopal episodes, however, there was no evidence of neurological diseases, including epilepsy and endocrine diseases. On admission, the blood test revealed a negative anion gap, hyperchloremia, and neutrophilic leukocytosis. Since one of the major causes of negative anion gaps with hyperchloremia is bromide intoxication, we measured the serum bromide level and found that it was significantly high at 21 mg/dl. Bromide is not normaly detectable in healthy indivisuals. Furthermore, 296 empty sheets of over-the-counter(O-T-C) analgesic tablets containing bromide (Naronace: O-T-C analgesic in Japan, contain bromovalerylurea 100 mg/ tablet) were found in her house, suggesting that she had bromide intoxication. Repeated syncopal episodes may have been due to this intoxication. These findings forced us to reconsider the diagnosis of pyoderma gangrenosum in this case, because bromide causes the same clinical symptoms as pyoderma. In addition, we realized neutrophilic leukocytosis and hyperchloremia had been associated with exacerbation of the clinical symptoms for 11 years. Therefore, we examined the bromide level of the serum samples collected from the past 10 years and found that the bromide level had been as high as 112.7 mg/dl. In conclusion, we diagnosed this case with bromoderma caused by O-T-C analgesic mimicking pyoderma gangrenosum. After the patient stop taking the analgesic, the pyoderma rapidly disappeared, and the white blood cell count and the serum chloride level returned to normal levels, with no recurrence, even after the discontinuation of prednisolone and cyclosporine.

